# Factors associated with the initiation of daily oral pre-exposure prophylaxis among adolescent girls and young women: findings from the Namibia DREAMS program

**DOI:** 10.3389/fpubh.2025.1665752

**Published:** 2025-09-17

**Authors:** Enos Moyo, Endalkachew Melese, Hadrian Mangwana, Simon Takawira, Rosalia Indongo, Bernadette Harases, Perseverance Moyo, Ernest Peresu, Kopano Robert, Tafadzwa Dzinamarira

**Affiliations:** ^1^School of Nursing and Public Health, College of Health Sciences, University of Kwa-Zulu Natal, Durban, South Africa; ^2^Project HOPE-The People-to-People Health Foundation, Inc., Windhoek, Namibia; ^3^Project HOPE Namibia, Windhoek, Namibia; ^4^Medical Centre Oshakati, Oshakati, Namibia; ^5^Centre for Health Systems Research and Development, University of Free State, Bloemfontein, South Africa; ^6^Faculty of Health, Humanities, and Social Sciences, School of Nursing, Welwitschia University, Windhoek, Namibia; ^7^Faculty of Health Sciences, School of Health Systems and Public Health, University of Pretoria, Pretoria, South Africa

**Keywords:** Determined, Resilient, Empowered, AIDS-Free, Mentored, and Safe project, Reducing HIV Vulnerability: Integrated Child and Youth Health project, project HOPE Namibia, PrEP initiation, determinants, adolescent girls and young women

## Abstract

**Background:**

Adolescent girls and young women (AGYW) aged 15–24 years in sub-Saharan Africa continue to face a significant risk of HIV acquisition. Oral pre-exposure prophylaxis (PrEP) can reduce the likelihood of HIV acquisition by more than 90% when adherence is optimal. The Determined, Resilient, Empowered, AIDS-Free, Mentored, and Safe (DREAMS) program, funded by PEPFAR/USAID and implemented by the Project HOPE Namibia (PHN)-led consortium, provided services in the Khomas, Oshikoto, Zambezi, and Oshana regions. The DREAMS program addresses factors that increase HIV vulnerability among AGYW. The objective of this secondary analysis of DREAMS program data is to assess the rate of daily oral PrEP initiation among AGYW aged 15–24, as well as the participants’ characteristics and HIV risk factors associated with PrEP initiation.

**Methods:**

The program’s target populations for PrEP included AGYW aged 15–24 years, who were at substantial risk for HIV, tested HIV-negative, and resided in the regions where the PHN-led consortium was implementing the DREAMS program. Site-level personnel utilized the District Health Information Software 2 (DHIS2) Android Application for regular data collection. Data from 2018 to 2024 were analyzed using IBM Statistical Package for Social Sciences (SPSS) version 29. Data analysis employed Chi-squared tests and binomial and multivariate logistic regression.

**Results:**

Among the 29,828 AGYW eligible for PrEP, 24,182 (81.1%) were newly initiated on PrEP. AGYW from Windhoek and Oshakati, those enrolled between 2018 and 2023, those with 1–2 biological children, and those who perceived themselves at risk of HIV were more likely to initiate PrEP. In contrast, participants from Omuthiya and Tsumeb, those unaware of their partners’ HIV status, those with HIV-positive partners during pregnancy or breastfeeding, those with recent or recurrent sexually transmitted infections, those engaging in sexual activity while under the influence of alcohol or drugs, and those with multiple or concurrent sexual partners were less likely to initiate PrEP.

**Conclusion:**

The findings highlight the need for improved HIV education in smaller urban and rural communities to reduce stigma and discrimination against individuals taking PrEP. Additionally, enhancing HIV education to increase risk perception among AGYW at substantial risk for HIV is essential.

## Introduction

The Joint United Nations Programme on HIV/AIDS (UNAIDS) statistics indicate that adolescent girls and young women (AGYW) aged 15–24 years in sub-Saharan Africa (SSA) continue to face a significant risk of HIV acquisition. Approximately 4,900 new infections are reported weekly among women in this age group worldwide (1). In 2022, approximately 27% of the 1.3 million new HIV cases globally were reported among individuals aged 15–24 years. Among adults aged 15 years and older, 66% of all new infections occurred in AGYW (2). Despite a 54% reduction in HIV incidence since its peak in 1996, the population of AGYW in SSA continues to be a critical demographic for HIV epidemic control (1).

The United Nations aims to eliminate the HIV/AIDS epidemic as a public health threat by the year 2030 (2). In 2023, the World Health Organization aimed to decrease new HIV infections to fewer than 370,000 by 2025 (2). A combination of HIV prevention measures should be implemented to achieve this target. Prevention measures can be categorized into behavioral, biomedical, and structural interventions. Biomedical interventions encompass condoms, voluntary medical male circumcision (VMMC), antiretroviral treatment as prevention (TasP), prevention of mother-to-child transmission (PMTCT), pre-exposure prophylaxis (PrEP), and post-exposure prophylaxis (PEP) (3).

PrEP involves the administration of antiretroviral medications to individuals who are HIV-negative and at significant risk, aimed at preventing HIV acquisition. In 2016, the WHO recommended oral PrEP for HIV prevention among all individuals at significant risk of HIV infection (4). This recommendation was based on a systematic review of 12 randomized controlled trials involving tenofovir disoproxil fumarate (TDF)-based oral PrEP (5). Oral PrEP can decrease the likelihood of HIV acquisition by more than 90% when adherence is optimal (4). The global population utilizing oral PrEP increased from approximately 200,000 in 2017 to 3.5 million in 2023. Nonetheless, this figure remains significantly below the global target of 21.2 million for 2025 (6).

Previous research has identified various factors associated with initiating PrEP among AGYW. A study conducted in South Africa indicated that a lack of knowledge regarding a partner’s HIV status, along with having an HIV-negative partner, enhances the probability of initiating PrEP (7). A South African study focusing on young women identified several factors that facilitate PrEP initiation among AGYW, including rural residence, having a casual partner, being married, never using contraceptive pills, and a partner age difference exceeding 10 years (8). A study involving pregnant and postpartum AGYW found no association between PrEP initiation and HIV risk perception (9). In a previous study conducted in Namibia, factors linked to increased PrEP initiation included early school dropout before Grade 12, having two or more sexual partners in the preceding 12 months, a history of concurrent sexual partners, and engagement in transactional sex (10). The Namibian study indicated that PrEP initiation was more probable among participants in the Zambezi region, and it was attributed to a high prevalence and incidence of HIV among AGYW in the region (10).

Namibia, with a population of approximately 3 million, has 9.7% of individuals aged 15–49 years living with HIV. The annual HIV incidence rate for individuals aged 15–49 years is 0.375%. Among people living with HIV in the country, 93% are aware of their status, 89% are receiving antiretroviral therapy (ART), and 87% of those on ART are virally suppressed (11). Among the younger populations, the HIV prevalence in AGYW between 15 and 24 years of age is 4.9%, while the HIV prevalence in adolescent boys and young men (ABYM) in the same age group is 2.2% (12). Adolescents and young people 15–24 years old account for 35% of all new HIV infections, with AGYW 15–24 years of age disproportionately affected. The HIV incidence among AGYW is 0.9%, while that among ABYM is 0.2% (12). The United States of America President’s Emergency Plan for AIDS Relief (PEPFAR) provided technical assistance to facilitate the incorporation of oral PrEP into Namibia’s national guidelines in 2016 (13).

PEPFAR and partners launched the Determined, Resilient, Empowered, AIDS-free, Mentored, and Safe (DREAMS) program, which is a public-private partnership designed to reduce the rate of HIV among AGYW in 15 countries, including Namibia (14). A consortium led by Project HOPE Namibia (PHN) successfully implemented the PEPFAR/USAID-funded DREAMS project from June 4, 2018, to July 31, 2023. PHN is currently leading a consortium to implement the follow-on activity, Reducing HIV Vulnerability: Integrated Child and Youth Health (Reach) PHN, awarded on July 31, 2023. Unlike its predecessor, Reach PHN includes DREAMS and Orphan and Vulnerable Children (OVC) components. There is limited literature on the uptake of PrEP among AGYW in Namibia and the factors associated with PrEP initiation. This manuscript presents the results of a secondary data analysis on the DREAMS program, implemented by the PHN-led consortia under the DREAMS project and Reach PHN, focusing on PrEP initiation among AGYW. The objective of the secondary data analysis was to assess the PrEP initiation rate among AGYW, the participants’ characteristics, and the HIV risk factors linked to PrEP initiation. The study’s findings are anticipated to guide the scale-up of PrEP services in Namibia.

## Methods

### Study design

We conducted a secondary data analysis of programmatic data collected from AGYW who received clinical services through the Namibia DREAMS program, implemented by the PHN-led consortiums under both the DREAMS project and Reach PHN, between 2018 and 2024.

### Program implementation setting

**Figure 1:**
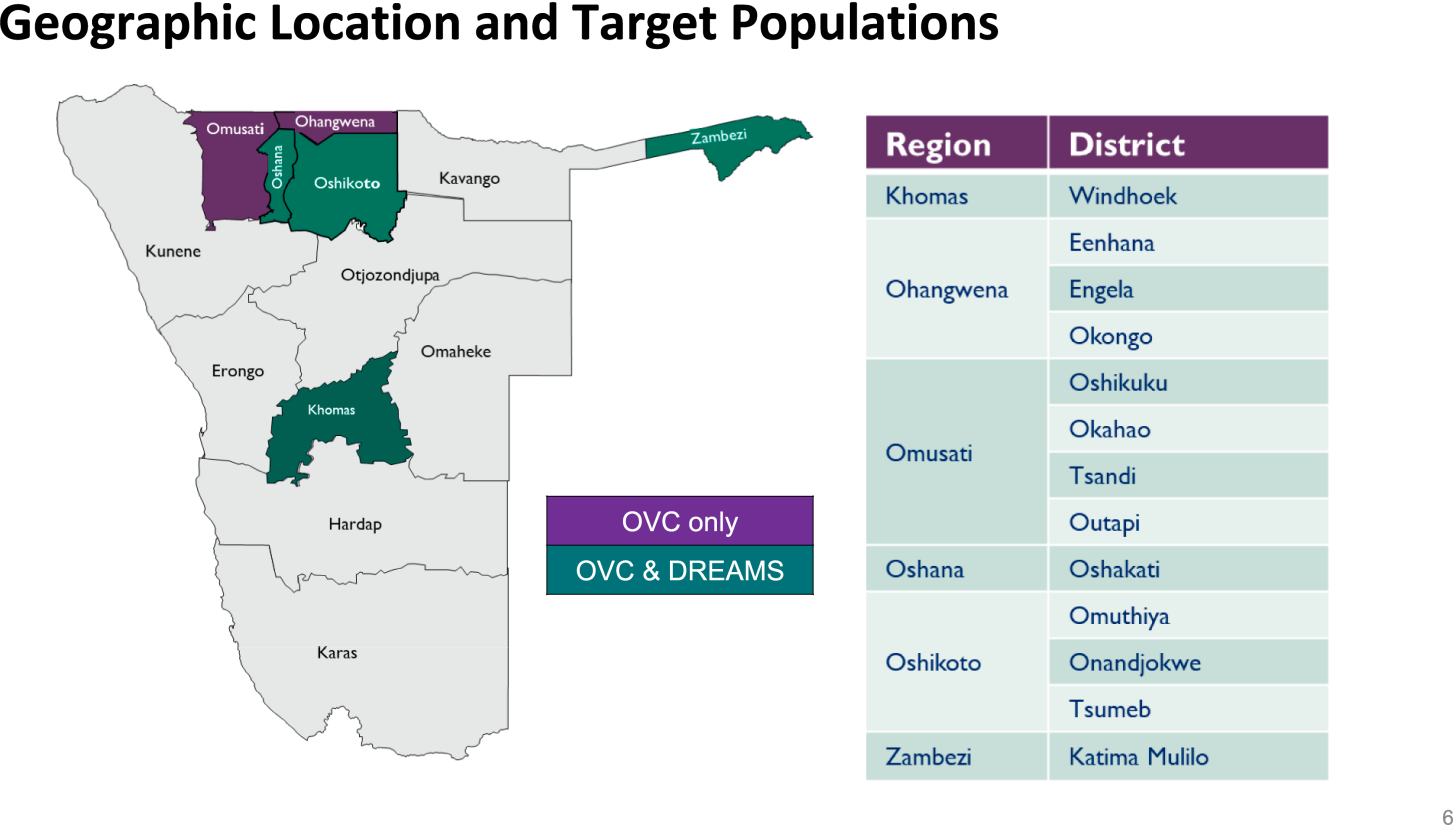
Geographical location and target populations of the PHN DREAMS program.

The PHN DREAMS program was implemented in Khomas, Oshikoto, and Zambezi regions, and Reach PHN expanded these services to Oshana. Socioeconomic disparities exist between regions. The majority of the 494,605 Khomas residents are female (51.3%), stay in urban areas (97%), and are single (72.5%). Five percent of Khomas residents have never had a formal education. In Khomas, 64% of households rely on wages and salaries, and approximately 5% live in poverty. The majority of Oshana’s 230,801 residents are female (53.8%), live in urban areas (53.2%), and are single (75.8%). About 6% of Oshana residents have never had a formal education. Nearly 40% of Oshana households rely on wages and salaries, while 21.1% are classified as poor. Oshikoto has 257,302 people, 50.5% of whom are women. Most Oshikoto residents are single (72.2%). About 18% of the population in Oshikoto lives in urban areas, and 12% have never had a formal education. Nearly 43% of Oshikoto households live in poverty, and 33.3% rely on wages and salaries. The Zambezi region’s 142,373 residents consist of 50.8% females. Over half (52.5%) of Zambezi residents are single. A third of the Zambezi residents stay in urban areas. Nearly 38% of Zambezi households rely on wages and salaries, and 39.3% live in poverty (15, 16). Figure 1 shows the regions and cities where the PHN DREAMS program was implemented.

### DREAMS intervention

The Dreams Intervention was discussed elsewhere (17). In summary, it addressed factors that increase HIV vulnerability among AGYW such as gender-based violence, economic exclusion, and limited access to health services. AGYW were grouped by age (10–12, 13–14, 15–19, and 20–24) and location into “safe spaces” when they joined the DREAMS program. Safe spaces were judgment-free, secure, and private locations where AGYW groups meet regularly under trained mentors’ guidance. For AGYW aged 10–12 and 13–24 years, a minimum of 16 and 19 sessions of HIV and violence prevention education, respectively, were delivered using the Window of Hope (WoH) curriculum (18). They also received social asset-building, HIV risk assessments, and screening for Gender-Based Violence (GBV) and Sexual and Reproductive Health (SRH) needs. AGYW aged 15–19 received a minimum of 10 sessions of HIV and violence prevention education using the My Future My Choice (MFMC) curriculum (18), along with social asset-building, risk assessments for HIV, and screening for GBV and SRH needs. Additionally, condom, PrEP, and contraceptive education was offered. AGYW aged 20–24 received similar interventions as those provided to the 15–19 age group. Secondary intervention requirements were assessed every 6 months. These interventions varied by age but included PrEP, post-violence care, contraceptive methods, economic strengthening interventions, and educational support.

### DREAMS approach to PrEP provision

The DREAMS program combined health facility-based delivery via adolescent and youth-friendly corners with community-based service delivery (fixed/mobile) through facility-linked outreach to provide nurse-led, comprehensive PrEP services. Private and confidential community-based PrEP service delivery was provided in schools, churches, community centers, and other chosen venues, as well as tents and trucks outside permanent community sites. AGYW could switch service delivery models during commencement or follow-up with the hybrid model.

DREAMS Nurses trained in PrEP used the National Risk Assessment and Clinical Eligibility Screening (RACES) tool to screen all sexually active girls aged 15–24 for PrEP eligibility at all service delivery points. Factors that were considered for PrEP eligibility include pregnancy or breastfeeding, an unknown HIV status of partner, being involved in transactional or intergenerational sex, recent STI (within the last 3 months) and/or having recurrent STIs, multiple and/or concurrent sexual partners, history of inconsistent or no condom use, being in a sero-discordant relationship where the partner’s viral load was unsuppressed, being in an abusive relationship, being a recurrent post-exposure prophylaxis (PEP) user, having a history of having sex while under the influence of alcohol or recreational drugs, and an individual strongly felt that she was at a substantial risk of HIV infection.

HIV Testing Services were provided to AGYW at high risk for HIV who wanted to utilize PrEP. Nurses collected and transmitted specimens for creatinine, HBSAg, RPR, and a baseline pregnancy test after a negative HIV rapid test. Same-day PrEP was started. If test results required further action, confirmation, or treatment, AGYW on PrEP was contacted. National guidelines guided follow-up visits and lab tests. The Ministry of Health and Social Services (MHSS) PrEP client record and the PHN-led consortium clinical register were used to record initiation and follow-up data.

### Data collection

PHN and its partners utilized a standardized data collection instrument to assess participants’ HIV status and risk, ensuring consistency and comparability across sites. Site-level personnel employed the District Health Information Software 2 (DHIS2) Android Application for systematic data collection from PrEP client records and clinical registers. Data on the sociodemographic characteristics and HIV risk factors of participants were extracted from the DREAMS participant records and the PrEP clients’ records. Data at the individual level were integrated from various sources utilizing the unique identification number assigned to DREAMS participants.

### Data quality assurance

The program’s data quality assurance is detailed in a separate publication (19). In summary, the digital system facilitated the automatic generation of unique identifier codes for each participant, implemented automated skip rules, and conducted validation checks for variables. Data quality assurance (DQA) mechanisms included periodic programmatic spot checks, desk reviews, data quality reviews, and field monitoring activities conducted by district and regional teams to ensure that reported data met minimum quality standards.

### Criteria for inclusion in data analysis

Among the 30,864 AGYW who received clinical services through the program and were naïve to PrEP from 2018 to 2024, we excluded those aged 10–14, as PrEP is contraindicated for this age group. After removing the 10–14-year-olds, 29,828 AGYW remained. Data analysis was conducted on this remaining group of 29,828 participants.

### Data analysis

Data were exported from DHIS2 to IBM Statistical Package for Social Sciences (SPSS) version 29 for analysis. Nominal and ordinal data were analyzed using descriptive statistics, including percentages and frequencies. Chi-square tests assessed the relationships between PrEP initiation and the characteristics of participants, along with their HIV risk factors. Statistically significant characteristics identified through Chi-square tests were analyzed using binomial logistic regression to assess the strength of their associations with PrEP initiation. Characteristics demonstrating statistically significant associations with PrEP initiation at a *p*-value below 0.05 in binomial logistic regression were subsequently used in multivariate logistic regression to ascertain the adjusted odds ratios.

### Ethical considerations

The DREAMS program’s ethical considerations are detailed in another publication (17). In summary, the program received ethical approval from the relevant Namibian ministries, and the confidentiality of the collected data was assured. Assent was sought from all minors, while their parents and guardians provided informed consent. All AGYW above the age of 18 provided informed consent before participation. No institutional review board ethical approval was necessary for this secondary data analysis since we used anonymous programmatic data.

## Results

### Characteristics of participants

Out of a total of 29,828 AGYW who were eligible for PrEP, the majority were in the age group 20–24 years (*n* = 17,201; 57.7%), less than 6 months in the program (*n* = 19,498; 65.4%), not using any family planning method (*n* = 21,394; 71.7), had not participated in the safe space interventions (*n* = 25,346; 85%), and had no biological children (*n* = 24,947; 83.6%). Supplementary information is in Table 1.

**Table 1:** Frequency distribution of characteristics of participants.

Characteristics	Frequency *n* (%)
Age group (years)	
15–19	12,627 (42.3)
20–24	17,201 (57.7)
District	
Katima	5,335 (17.9)
Omuthiya	4,035 (13.5)
Onandjokwe	4,549 (15.3)
Oshakati	1,437 (4.8)
Tsumeb	2,501 (8.4)
Windhoek	11,971 (40.1)
Year of enrolment	
2018	439 (1.5)
2019	2,851 (9.6)
2020	8,413 (28.2)
2021	7,270 (24.4)
2022	4,671 (15.7)
2023	2,031 (6.7)
2024	4,153 (13.9)
Months in the program	
≤6 months	19,498 (65.4)
7–12 months	2,182 (7.3)
13–24 months	3,182 (10.6)
25–36 months	1,807 (6.1)
>36 months	3,159 (10.6)
Using family planning method	
Yes	8,434 (28.3)
No	21,394 (71.7)
Pregnant or breastfeeding	
Pregnant	66 (0.2)
Breastfeeding	881 (3.0)
Neither breastfeeding nor pregnant	1,996 (6.7)
Missing	26,885 (90.1)
Participated in safe space interventions	
Yes	4,482 (15.0)
No	25,346 (85.0)
Marital status	
Single	9,097 (30.5)
Married	58 (0.2)
Missing	20,673 (69.3)
Number of biological children	
0	24,947 (83.6)
1–2	4,647 (15.6)
≥3	234 (0.8)
PrEP initiation	
Yes	24,182 (81.1)
No	5,646 (18.9)

### HIV risk factors among AGYW eligible for PrEP

The majority of the participants did not know their partners’ HIV status (*n* = 15,752; 52.8%), did not use condoms or inconsistently used them (*n* = 19,234; 64.5%), and considered themselves at risk of HIV (*n* = 19,086; 64%). However, the majority of the participants either did not have or did not report the following risk factors: recent or recurrent STIs (*n* = 29,579; 99.2%), sex under the influence of alcohol or drugs (*n* = 29,639; 99.4%), and multiple or recurrent sexual partners (*n* = 29,325; 98.3%). More details are in Table 2.

**Table 2:** Frequency distribution of HIV risk factors among participants.

Characteristics	Frequency *n* (%)
Unknown partner’s HIV status	
Yes	15,752 (52.8)
No	14,076 (47.2)
Partner is HIV-positive and not on antiretroviral therapy	
Yes	47 (0.2)
No	29,781 (99.8)
Partner with detectable viral load	
Yes	120 (0.4)
No	29,708 (99.6)
Has an HIV-positive partner and is pregnant or breastfeeding	
Yes	51 (0.2)
No	29,777 (99.8)
Inconsistent or no condom use	
Yes	19,234 (64.5)
No	10,594 (35.5)
Recent or recurrent STIs	
Yes	249 (0.8)
No	29,579 (99.2)
Recurrent post-exposure prophylaxis (PEP) use	
Yes	34 (0.1)
No	29,794 (99.9)
Having had sex under the influence of alcohol or drugs	
Yes	189 (0.6)
No	29,639 (99.4)
A victim of sexual violence	
Yes	64 (0.2)
No	29,764 (99.8)
Has multiple or recurrent sexual partners	
Yes	503 (1.7)
No	29,325 (98.3)
Considers self at risk of HIV	
Yes	19,086 (64.0)
No	10,742 (36.0)

### PrEP initiation rate among participants

**Figure 2:**
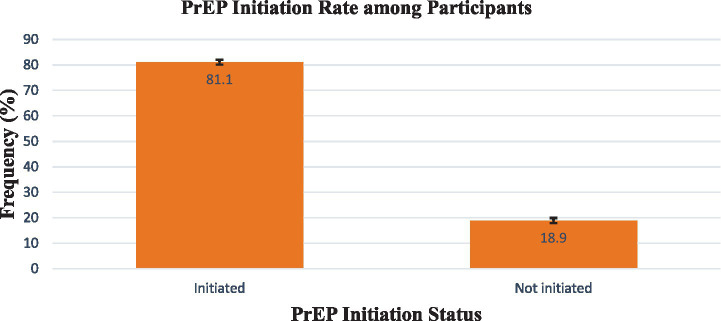
PrEP initiation rate among participants.

Overall, 24,182 eligible participants (81.1%) were initiated on PrEP, 95% confidence interval (CI) (80.7–81.5%), while 5,646 (18.9%) were not initiated, 95% CI (18.5–19.3%). More details are in Figure 2.

### Participants’ characteristics that are associated with PrEP initiation

The Chi-square tests revealed associations between PrEP initiation and district, year of enrolment, the number of months in the program, safe space participation status, and the number of biological children (*p* < 0.05). Participants in Omuthiya and Tsumeb had lower chances of initiating PrEP than those in Katima Mulilo, adjusted odds ratio (AOR) = 0.32, 95% CI (0.29–0.36), and AOR = 0.30, 95% CI (0.26–0.33), respectively. However, participants in Oshakati and Windhoek were statistically significantly more likely to initiate PrEP than those in Katima Mulilo, AOR = 7.41, 95% CI (5.59–5.99), AOR = 21.70, 95% CI (11.64–40.43), AOR = 8.60, 95% CI (3.16–23.40), and AOR = 8.76, 95% CI (5.99–9.84), respectively. Participants enrolled in 2019–2023 were statistically significantly more likely to initiate PrEP than those enrolled in 2024. Participants who had more than 6 months in the program were statistically significantly less likely to initiate PrEP than those with less than 6 months. Safe space participation decreased the likelihood of PrEP initiation (AOR) = 0.76; 95% CI (0.70–0.83). Single participants were more likely to initiate PrEP than married ones, crude odds ratio (COR) = 1.78, 95% CI (1.01–3.18). However, marital status was not included in the multinomial analysis due to missing data for most participants. Participants with 1–2 children were more likely to initiate PrEP compared to those without children, AOR = 1.13, 95% CI (1.03–1.25). More details are in Table 3.

**Table 3:** Associations between PrEP initiation and characteristics of participants.

Characteristics	Crude odds ratios	95% CI^*^	Adjusted^**^ odds ratios	95% CI^*^	Chi-square test *p*-value
Age group (years)					0.97
15–19	NI	NI	NI	NI	
20–24	NI	NI	NI	NI	
District					**<0.01**
Katima Mulilo	Reference	Reference	Reference	Reference	
Omuthiya	**0.42**	**0.38–0.46**	**0.32**	**0.29–0.36**	
Onandjokwe	**1.21**	**1.08–1.34**	1.06	0.96–1.19	
Oshakati	**5.50**	**4.19–7.24**	**7.41**	**5.59–9.84**	
Tsumeb	**0.40**	**0.36–0.45**	**0.30**	**0.26–0.33**	
Windhoek	**1.54**	**1.40–1.66**	**1.34**	**1.23–1.47**	
Year of enrolment					**<0.01**
2018	**1.52**	**1.13–2.04**	**7.15**	**5.21–9.81**	
2019	1.06	0.93–1.20	**4.76**	**4.06–5.57**	
2020	**0.82**	**0.75–0.91**	**2.46**	**2.18–2.78**	
2021	**0.73**	**0.66–0.81**	**1.80**	**1.60–2.02**	
2022	**1.25**	**1.12–1.41**	**2.30**	**2.03–2.60**	
2023	1.05	0.91–1.20	**1.82**	**1.56–2.11**	
2024	Reference	Reference	Reference	Reference	
Months in the program					**<0.01**
≤6 months	Reference	Reference	Reference	Reference	
7–12 months	**0.72**	**0.65–0.80**	**0.74**	**0.66–0.84**	
13–24 months	**0.69**	**0.62–0.74**	**0.66**	**0.59–0.73**	
25–36 months	**0.52**	**0.47–0.59**	**0.43**	**0.38–0.48**	
>36 months	**0.56**	**0.52–0.62**	**0.33**	**0.30–0.37**	
Using a family planning method					0.22
Yes	NI	NI	NI	NI	
No	NI	NI	NI	NI	
Pregnant or breastfeeding					0.18
Pregnant	NI	NI	NI	NI	
Breastfeeding	NI	NI	NI	NI	
Neither	NI	NI	NI	NI	
Participated in safe space HIV prevention sessions					**<0.01**
Yes	**0.74**	**0.69–0.80**	**0.76**	**0.70–0.83**	
No	Reference	Reference	NI	NI	
Marital status					**0.05**
Single	**1.78**	**1.01–3.18**	NI	NI	
Married	Reference	Reference	NI	NI	
Number of biological children					**0.03**
0	Reference	Reference	Reference	Reference	
1–2	**1.12**	**1.03–1.21**	**1.13**	**1.03–1.25**	
≥3	1.03	0.74–1.43	1.25	0.89–1.77	

NC, Not computed; NI, Not included; ^*^CI is the 95% confidence interval; ^**^Adjusted for district, year of enrolment, number of months in the program, participation in safe space HIV prevention sessions, and the number of biological children. Bold values mean the results are statistically significant.

### HIV risk factors associated with PrEP initiation

The Chi-square tests revealed associations between PrEP initiation and several risk factors, including not knowing a partner’s HIV status, the partner’s viral load status, the participant’s pregnancy or breastfeeding status, having an HIV-positive partner while pregnant or breastfeeding, recent or recurrent STIs, engaging in sex under the influence of alcohol or drugs, having multiple or concurrent sexual partners, and considering self at risk of HIV. In contrast, the Chi-square tests did not reveal any associations between PrEP initiation and inconsistent or no condom use and recurrent PEP use. Participants who did not know their partners’ HIV status, whose partners were HIV-positive when they were pregnant or breastfeeding, had recent or recurrent STIs, who had sex under the influence of alcohol or drugs, and who had multiple or concurrent sexual partners were statistically significantly less likely to initiate PrEP, AOR = 0.74, 95% CI (0.69–0.79), AOR = 0.44, 95% CI (0.24–0.80), AOR = 0.57, 95% CI (0.42–0.76), AOR = 0.56, 95% CI (0.40–0.79), and AOR = 0.58, 95% CI (0.47–0.72), respectively. However, participants who considered themselves at risk of HIV were statistically significantly more likely to initiate PrEP, AOR = 5.05, 95% CI (4.73–5.39). More information is in Table 4.

**Table 4:** Associations between PrEP initiation and risk factors of participants.

Characteristics	Crude odds ratios	95% CI^*^	Adjusted^**^ odds ratios	95% CI^*^	Chi-square test *p*-value
Partner’s HIV status is unknown					**<0.01**
Yes	**1.26**	**1.19–1.34**	**0.74**	**0.69–0.79**	
No	Reference	Reference	Reference	Reference	
Partner is HIV-positive and not on antiretroviral therapy					0.68
Yes	NC	NC	NI	NI	
No	NC	NC	NI	NI	
Partner with detectable viral load					**0.02**
Yes	**0.61**	**0.41–0.92**	0.69	0.45–1.05	
No	Reference	Reference	Reference	Reference	
Has an HIV-positive partner and is pregnant or breastfeeding					**<0.01**
Yes	**0.33**	**0.19–0.58**	**0.44**	**0.24–0.80**	
No	Reference	Reference	Reference	Reference	
Inconsistent or no condom use					0.75
Yes	NC	NC	NI	NI	
No	NC	NC	NI	NI	
Recent or recurrent STIs					**<0.01**
Yes	**0.51**	**0.39–0.67**	**0.57**	**0.42–0.76**	
No	Reference	Reference	Reference	Reference	
Recurrent post-exposure prophylaxis (PEP) use					0.49
Yes	NC	NC	NI	NI	
No	NC	NC	NI	NI	
Having had sex under the influence of alcohol or drugs					**<0.01**
Yes	**0.54**	**0.39–0.74**	**0.56**	**0.40–0.79**	
No	Reference	Reference	Reference	Reference	
Has multiple or concurrent sexual partners					**<0.01**
Yes	**0.70**	**0.57–0.86**	**0.58**	**0.47–0.72**	
No	Reference	Reference	Reference	Reference	
Considers self at risk of HIV					**<0.01**
Yes	**4.51**	**4.24–4.80**	**5.05**	**4.73–5.39**	
No	Reference	Reference	Reference	Reference	

NC, Not computed; NI, Not included; ^*^CI is the 95% confidence interval; ^**^Adjusted for knowledge of partner’s HIV status, partner’s viral load status, HIV-positive partner while pregnant or breastfeeding, recent or recurrent STIs, having had sex under the influence of alcohol or drugs, having multiple or concurrent sexual partners, and consideration of HIV self-risk. Bold values mean the results are statistically significant.

## Discussion

The programs’ findings indicated that the PrEP initiation rate among eligible AGYW was 81.1%. Participants in Windhoek and Oshakati, those who enrolled from 2018 to 2023, those who had 1–2 biological children, or perceived themselves at risk of HIV, were more likely to initiate PrEP. Conversely, participants in Omuthiya and Tsumeb, those who had been in the program for over 6 months, those unaware of their partners’ HIV status, those with HIV-positive partners while pregnant or breastfeeding, those with recent or recurrent STIs, and those engaging in sexual activity while under the influence of alcohol or drugs, those with multiple or concurrent sexual partners, and those who participated in safe space HIV prevention sessions demonstrated a lower likelihood of initiating PrEP.

The program’s 81.1% PrEP initiation rate is significantly higher than the 4% reported in a Kenyan study conducted in family planning clinics (20) and the 36.9% reported in a DREAMS program study conducted in Namibia (10). The high PrEP initiation in the current program can be ascribed to the DREAMS eligibility criteria, which specifically targeted AGYW at heightened risk of HIV. The project activities, including the provision of HIV education and youth-friendly services, likely improved AGYW’s understanding of HIV, resulting in a heightened perception of HIV risk among this group. However, it is essential to note that during DQA and supervision, we discovered that in some districts, the RACES tool was only completed for AGYW who were initiated on PrEP, which might inadvertently have resulted in a higher PrEP initiation rate. Participants in Omuthiya and Tsumeb exhibited a lower likelihood of initiating PrEP than those in Katima Mulilo. In contrast, participants in Windhoek and Oshakati demonstrated a higher likelihood of initiating PrEP than their counterparts in Katima Mulilo. The findings differ from those of a comparable project conducted by the International Training and Education Center for Health (I-TECH), which indicated that participants in the Zambezi region had a higher likelihood of initiating PrEP than those in the Khomas region (10). Our findings may be attributed to more participants in the program for less than or equal to 6 months in Windhoek. The expectation in the program was that AGYW who had been in the program for a longer period would be more likely to reduce their risk behavior, resulting in them not requiring PrEP. Additionally, AGYW from smaller towns or more rural communities might have been concerned about being seen while accessing PrEP services by their peers or family members, potentially leading to stigmatization. There is a belief in some African communities that AGYW who take oral PrEP have multiple sexual partners (21). Regional variations in HIV knowledge levels may also exist, influenced by differing exposure to mass media and the Internet. Insufficient knowledge regarding HIV prevention can lead to a diminished perception of HIV risk (22). The findings underscore the need to improve HIV education in smaller urban and rural communities to reduce stigma and discrimination against those taking PrEP.

The present study found that participants with more than 6 months of involvement in the programs were less likely to initiate PrEP than those with 6 months or less of participation. Additionally, AGYW engaged in safe space interventions were also less likely to start PrEP. These findings may be related to reduced risky behavior among these participants, likely due to the HIV risk reduction interventions provided by the DREAMS program. Reinforcing HIV prevention messaging is crucial, even for long-term program participants or those engaged in safe space interventions, to ensure continuous evaluation of their HIV risk and the adoption of necessary precautions. However, it is important to note that the duration of program participation is measured from the day an AGYW is enrolled in the DREAMS program without accounting for their actual participation status (Active vs. Inactive). As a result, a considerable number of AGYW with over 6 months of program participation may not have been active participants for the entire duration. Participants with multiple or concurrent partners were less likely to initiate PrEP. The findings differ from an earlier Namibian study, indicating that individuals who had multiple or concurrent sexual partners were more inclined to initiate PrEP (10). The findings of this study may indicate a low HIV risk perception among AGYW who had multiple or concurrent sexual relationships. It is important to note that AGYW with a high epidemiological risk of HIV may not possess a corresponding high-risk perception (23). The study revealed that AGYW perceiving themselves as at risk for HIV were approximately four times more likely to commence PrEP. These are likely AGYW who have recognized their risk and requested PrEP but are uncomfortable disclosing the specific risk factor.

AGYW whose partners were HIV-positive while pregnant or breastfeeding had a lower likelihood of initiating PrEP than those who were neither pregnant nor breastfeeding. We would have expected pregnant or breastfeeding AGYW to show an increased motivation to initiate PrEP, driven by the desire to safeguard their health and ensure the birth of HIV-negative children (19). This may be due to concerns about the potential side effects of PrEP medications on the fetus or infant. Addressing misconceptions about the side effects of PrEP is essential to increase its initiation among pregnant and breastfeeding AGYW. Also of concern is that participants who were at high risk, such as having a partner with an unknown HIV status, recent or recurrent STIs, and having sex under the influence of alcohol or drugs, were less likely to initiate PrEP. The findings may be attributed to a low HIV-risk perception. The findings highlight the need for enhanced HIV prevention education for high-risk AGYW to increase risk perception. Additionally, user-friendly services should be strengthened at all PrEP service delivery sites.

The use of family planning methods was notably low among AGYW eligible for PrEP, likely due to frequent stockouts of commodities and a lack of awareness that PrEP does not prevent pregnancy or other STIs. These findings underscore the need for improved sexual and reproductive health education and urgent interventions to ensure commodity security.

This study’s strength lies in its utilization of a large sample size to assess associations between PrEP initiation and participants’ characteristics and HIV risk factors, thereby enhancing the representativeness of the findings within the target population. The digital data collection minimized the likelihood of errors and facilitated automated data cleaning. Data processing and cleaning were conducted, allowing for the identification and correction of errors in near real time. Another strength is the implementation of validation rules designed to notify front-end users during data entry, thereby preventing incomplete and inaccurate submissions. Furthermore, periodic training, site-level mentorship, supportive supervision, and data quality assessments were implemented. However, the responses were self-reported, so they might have been influenced by social desirability bias.

## Conclusion

AGYW from SSA aged 15–24 years are at a significant risk of HIV acquisition. Oral daily PrEP can decrease the likelihood of HIV acquisition by more than 90% when adherence is maintained. The programs’ findings indicated that the PrEP initiation rate among eligible AGYW was 81.1%. The PrEP initiation rate among eligible AGYW is affected by various factors. AGYW from Windhoek and Oshakati, those enrolled between 2018 and 2023, those with 1–2 biological children, and those who perceived themselves at risk of HIV were more likely to initiate PrEP. In contrast, participants from Omuthiya and Tsumeb, those unaware of their partners’ HIV status, those with HIV-positive partners during pregnancy or breastfeeding, those with recent or recurrent STIs, those engaging in sexual activity while under the influence of alcohol or drugs, those with multiple or concurrent sexual partners, and those who participated in safe space HIV interventions were less likely to initiate PrEP. The findings highlight the need for improved HIV education in smaller urban and rural communities to reduce stigma and discrimination against individuals taking PrEP. Additionally, enhancing HIV education to increase risk perception among AGYW at substantial risk for HIV is essential. The use of family planning methods was notably low among AGYW eligible for PrEP, highlighting the need for improved SRH education and urgent interventions to ensure commodity security.

## Data Availability

The original contributions presented in the study are included in the article/supplementary material, further inquiries can be directed to the corresponding author.
